# Efflux Pump Antibiotic Binding Site Mutations Are Associated with Azithromycin Nonsusceptibility in Clinical Neisseria gonorrhoeae Isolates

**DOI:** 10.1128/mBio.01509-20

**Published:** 2020-08-25

**Authors:** Kevin C. Ma, Tatum D. Mortimer, Yonatan H. Grad

**Affiliations:** a Department of Immunology and Infectious Diseases, Harvard T. H. Chan School of Public Health, Boston, Massachusetts, USA; b Division of Infectious Diseases, Brigham and Women’s Hospital and Harvard Medical School, Boston, Massachusetts, USA; Emory University School of Medicine; Harvard Medical School

## LETTER

Lyu and Moseng et al. used cryo-electron microscopy to characterize key residues involved in drug binding by mosaic-like MtrD efflux pump alleles in Neisseria gonorrhoeae (1). Isogenic experiments introducing key MtrD substitutions R714G and K823E increased macrolide MICs, leading the authors to predict that nonmosaic MtrD “gonococcal strains bearing both the *mtrR* promoter and amino acid changes at MtrD positions 714 or 823 could lead to clinically significant levels of Azi nonsusceptibility resistance.” We tested this hypothesis by analyzing a global meta-analysis collection of 4,852 N. gonorrhoeae genomes (2). In support of their prediction, we identified clinical isolates with novel nonmosaic MtrD drug binding site substitutions across multiple genetic backgrounds associated with elevated azithromycin MICs (Table 1).

**TABLE 1:** MtrD substitution strains, associated metadata, and resistance allele genotypes^*a*^

SRA accession no.	Reference	AZI MIC (μg/ml)	MtrD allele	Cluster^*b*^	*mtrR* promoter	RplD G70 allele	23S rRNA	*penA* allele
ERR1469714	9	1	R714H	1	Adel	WT	WT	XXXIV
ERR1528327	9	1	R714H	2	Adel	WT	WT	XXXIV
ERR1514686	9	NA	R714H	2	Adel	WT	WT	XXXIV
ERR1469709	9	1	R714H	1	Adel	WT	WT	XXXIV
SRR1661243	10	1	R714H	3	Adel	WT	WT	Nonmosaic
SRR2736280	11	2	R714H	NA	Adel	WT	WT	XXXIV
SRR2736175	11	2	R714H	3	Adel	WT	WT	Nonmosaic
SRR2736167	11	2	R714H	3	Adel	WT	WT	Nonmosaic
ERR349976	12	0.19	R714H	NA	WT	WT	WT	Nonmosaic
ERR854880	13	4	R714L	4	Adel	WT	WT	Nonmosaic
ERR855125	13	4	R714L	4	Adel	WT	WT	Nonmosaic
ERR855232	13	0.5	R714C	NA	Adel	WT	WT	XXXIV
ERR363653	12	0.75	K823E	NA	Adel	WT	WT	Nonmosaic
ERR855395	13	8	K823E	NA	Adel	R	WT	XXXIV
ERR855128	13	2	K823E	NA	Adel	WT	WT	Nonmosaic
ERR1067793	13	2	K823E	NA	Adel	WT	WT	Nonmosaic
SRR2736213	11	2	K823E	NA	Adel	S	WT	Nonmosaic
SRR2736281	11	2	K823E	NA	Adel	WT	WT	Nonmosaic
SRR2736124	11	2	K823N	NA	Adel	WT	WT	Nonmosaic

a Abbreviations: AZI, azithromycin; NA, not available; Adel, A deletion in 13 bp inverted repeat; WT, wild type.

b Cluster number corresponds to cluster number labels in the Fig. 1b phylogeny.

Of the 4,852 isolates, 12 isolates contained nonsynonymous mutations at position R714 to amino acid H, L, or C and 7 isolates contained K823 mutations to E or N in the nonmosaic MtrD background. We did not observe substitutions at positions 174, 669, 821, and 825, in line with the authors’ demonstration that isogenic mutants at these codons had identical or lowered macrolide MICs. The azithromycin geometric mean MICs of the clinical isolates with mutations at R714 and K823 were 1.25 μg/ml and 2.12 μg/ml, respectively, both of which are above the CLSI azithromycin nonsusceptibility threshold (Fig. 1a). There was a significant difference in mean MIC distributions comparing MtrD substitution strains with genetically matched controls (*P* = 0.0008, mean log_2_ MIC difference = 1.86, paired-sample Wilcoxon test; see Table S1 in the supplemental material). There was also a significant difference in mean MIC distributions for ceftriaxone (*P* = 0.045, mean log_2_ MIC difference = 0.56) but not for ciprofloxacin (*P* = 0.62).

**FIG 1:**
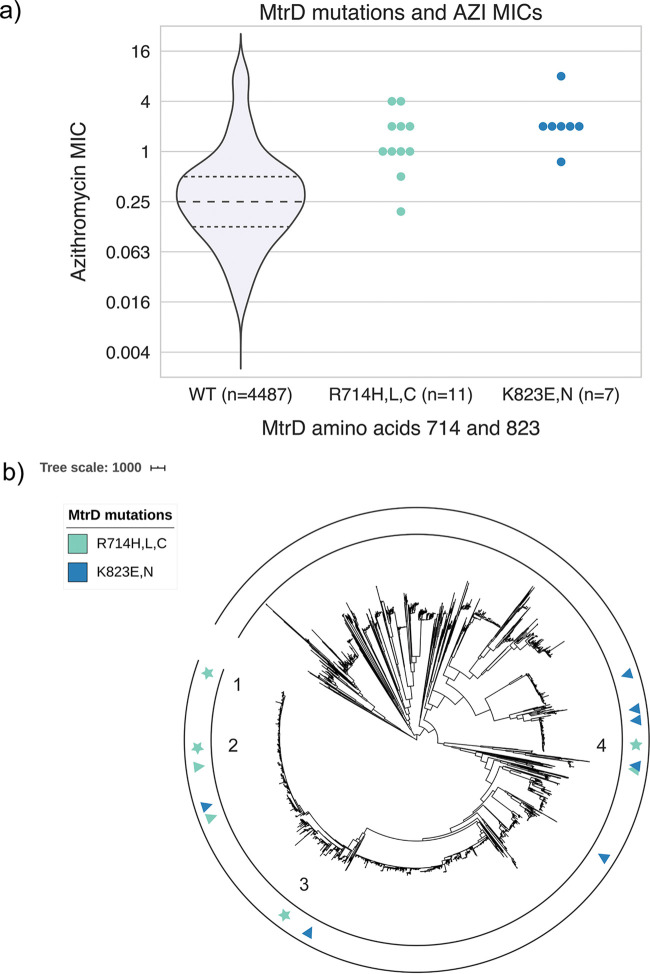
MtrD mutations associated with increased azithromycin MICs have emerged across the N. gonorrhoeae phylogeny. (a) Comparison of AZI MIC distributions for strains with and without nonmosaic MtrD substitutions at R714 and K823 and (b) phylogenetic distribution of MtrD substitution strains in a recombination-corrected phylogeny of the 4,852 strains from the global meta-analysis collection. In panel b, triangles indicate singleton strains and stars indicate clusters of two or more strains; cluster number labels correspond to cluster labels in Table 1.

10.1128/mBio.01509-20.1 TABLE S1 Comparison of AZI MICs of MtrD substitution strains and their nearest neighbors. After log transforming AZI MICs, statistical significance was assessed using a paired-sample Wilcoxon test. Download Table S1, DOCX file, 0.01 MB. Copyright © 2020 Ma et al. 2020 Ma et al. This content is distributed under the terms of the Creative Commons Attribution 4.0 International license.

Nearly all MtrD substitution strains contained *mtrR* promoter mutations that increase MtrCDE pump expression (Table 1) (3). The isolate with an MtrD R714H mutation and the lowest observed azithromycin MIC of 0.19 μg/ml did not have an *mtrR* promoter mutation, consistent with epistasis across the *mtrRCDE* operon (4). Contributions from ribosomal mutations can also synergistically increase macrolide resistance: the isolate with an MtrD K823E substitution and the highest observed azithromycin MIC of 8.0 μg/ml contained an RplD G70S mutation previously implicated in macrolide resistance (5). Seven MtrD isolates also had mosaic *penA* XXXIV alleles conferring cephalosporin reduced susceptibility, indicating a potential route to dual therapy resistance.

MtrD R714 and MtrD K823 substitutions were each acquired seven times across the phylogeny, suggesting that acquisition of the mutation is possible in different genetic backgrounds (Fig. 1b). Four of the MtrD K823 acquisitions were associated with more than one isolate descending from the same ancestor, suggesting that these strains are successfully transmitted. In line with this, nonrecombinant single nucleotide polymorphism (SNP) distances between isolates in each of the four clusters were all below 18 SNPs, with 3/4 clusters below the 10-SNP cutoff previously used as evidence for defining a transmission cluster (6, 7).

Complementing the experimental and structural biology approach taken by Lyu and Moseng et al. (1), we demonstrated using genomics that clinical isolates have acquired novel MtrD binding site mutations which, in combination with *mtrR* promoter and RplD mutations, can result in azithromycin nonsusceptibility. As azithromycin-resistant strains have been growing in prevalence (8), our data support the inclusion of MtrD binding site residues in future genomic surveillance and genotype-to-phenotype diagnostics and modeling studies for characterizing gonococcal resistance.

### Data availability.

All code, metadata, and intermediate analyses files to replicate analyses are available at https://github.com/gradlab/mtrD-resistance/. An interactive and downloadable version of the phylogeny is hosted at https://itol.embl.de/tree/1281032416307421591107815.

10.1128/mBio.01509-20.2 TEXT S1 Supplemental methods for the identification of MtrD substitutions and statistical testing of MIC differences between MtrD substitution strains and their nearest neighbors. Download Text S1, DOCX file, 0.02 MB. Copyright © 2020 Ma et al. 2020 Ma et al. This content is distributed under the terms of the Creative Commons Attribution 4.0 International license.
