# Supplementary material for: Efflux Pump Antibiotic Binding Site Mutations Are Associated with Azithromycin Nonsusceptibility in Clinical Neisseria gonorrhoeae Isolates
Source: mBio. 2020 Aug 25;11(4):e01509-20. doi: 10.1128/mBio.01509-20 (PMC7448274; doi:10.1128/mBio.01509-20)
Supplement: TABLE S1 [file mBio.01509-20-st001.docx]

| **MtrD substitution strain** | **MtrD Mutation** | **Substitution Strain AZI MIC (µg/mL)** | **Matched MtrD WT strain** | **WT Strain AZI MIC (µg/mL)** | **Non-recombinant SNP distance** |
| --- | --- | --- | --- | --- | --- |
| SRR1661243 | mtrD_714 | 1 | SRR1661226 | 0.25 | 4 |
| SRR2736124 | mtrD_823 | 2 | SRR2736094 | 2 | 7 |
| SRR2736167 | mtrD_714 | 2 | SRR1661226 | 0.25 | 15 |
| SRR2736175 | mtrD_714 | 2 | SRR1661226 | 0.25 | 5 |
| SRR2736213 | mtrD_823 | 2 | DRR124869 | 0.5 | 145 |
| SRR2736280 | mtrD_714 | 2 | ERR855279 | 1 | 17 |
| SRR2736281 | mtrD_823 | 2 | ERR855005 | 0.125 | 12 |
| ERR855128 | mtrD_823 | 2 | ERR1560878 | 0.125 | 106 |
| ERR1067793 | mtrD_823 | 2 | ERR1560869 | 1.5 | 22 |
| ERR855395 | mtrD_823 | 8 | ERR191732 | 1 | 29 |
| ERR855232 | mtrD_714 | 0.5 | ERR854884 | 0.5 | 9 |
| ERR854880 | mtrD_714 | 4 | ERR1471127 | 0.25 | 24 |
| ERR855125 | mtrD_714 | 4 | ERR1560869 | 1.5 | 28 |
| ERR1469709 | mtrD_714 | 1 | ERR1528280 | 0.25 | 11 |
| ERR1469714 | mtrD_714 | 1 | ERR1528280 | 0.25 | 11 |
| ERR1514686 | mtrD_714 | NA | ERR1560893 | 0.5 | 31 |
| ERR1528327 | mtrD_714 | 1 | ERR1560893 | 0.5 | 22 |
| ERR349976 | mtrD_714 | 0.19 | ERR1560863 | 0.38 | 772 |
| ERR363653 | mtrD_823 | 0.75 | ERR363634 | 0.25 | 71 |

**Supplementary Table S1 – Comparison of AZI MICs of MtrD substitution strains and their nearest neighbors.** After log­ transforming AZI MICs, statistical significance was assessed using a paired samples Wilcoxon test.
