# Supplementary material for: Efflux Pump Antibiotic Binding Site Mutations Are Associated with Azithromycin Nonsusceptibility in Clinical Neisseria gonorrhoeae Isolates
Source: mBio. 2020 Aug 25;11(4):e01509-20. doi: 10.1128/mBio.01509-20 (PMC7448274; doi:10.1128/mBio.01509-20)
Supplement: TEXT S1 [file mBio.01509-20-s0001.docx]

**Supplementary Text S1 – Supplementary methods for the identification of MtrD substitutions and statistical testing of MIC differences between MtrD substitution strains and their nearest neighbors.**

MtrD substitutions: We ran BLASTn on the assemblies using an *mtrD* reference sequence from gonococcal strain FA1090 (Genbank accession: NC_002946.2) and filtered out hits with lower than 99% identity to remove mosaic *mtrD* sequences. LOF alleles were also removed by identifying strains with predicted peptides 90% or shorter than the FA1090 allele. *mtrD* sequences were aligned using MAFFT (version 7.450) (1), translated, and diversity at residues 174, 669, 714, 821, 823, and 825 was characterized using Python (version 3.6.5) and Biopython (version 1.69) (2).

Nearest neighbor analysis: We calculated non-recombinant SNP distances using snp-dists (version 0.6.3, <https://github.com/tseemann/snp-dists>) from a recombination masked alignment of polymorphisms produced by a previous analysis of recombination using Gubbins (version 2.3.4) (3). For each strain with a MtrD mutation at codon 714 or 823, we identified the most closely related strain in the dataset without mutations at these positions and with available azithromycin MICs. We used a paired samples Wilcoxon test to test for significant differences between log transformed azithromycin MICs and their nearest neighbor in R (version 3.6.1). Non-recombinant SNP distances were also used to identify genetic distance between MtrD substitution strains within clusters.

**Supplementary references**

1. Katoh K, Misawa K, Kuma K, Miyata T. 2002. MAFFT: a novel method for rapid multiple sequence alignment based on fast Fourier transform. Nucleic Acids Res 30:3059-66.

2. Cock PJ, Antao T, Chang JT, Chapman BA, Cox CJ, Dalke A, Friedberg I, Hamelryck T, Kauff F, Wilczynski B, de Hoon MJ. 2009. Biopython: freely available Python tools for computational molecular biology and bioinformatics. Bioinformatics 25:1422-3.

3. Croucher NJ, Page AJ, Connor TR, Delaney AJ, Keane JA, Bentley SD, Parkhill J, Harris SR. 2015. Rapid phylogenetic analysis of large samples of recombinant bacterial whole genome sequences using Gubbins. Nucleic Acids Res 43:e15.
